# SCSIM: Jointly simulating correlated single-cell and bulk next-generation DNA sequencing data

**DOI:** 10.1186/s12859-020-03550-1

**Published:** 2020-05-26

**Authors:** Collin Giguere, Harsh Vardhan Dubey, Vishal Kumar Sarsani, Hachem Saddiki, Shai He, Patrick Flaherty

**Affiliations:** 1 Department of Mathematics & Statistics, University of Massachusetts Amherst, 710 N. Pleasant St., Amherst, 01003 USA; 2 School of Public Health, University of Massachusetts Amherst, Amherst, 01003 USA

**Keywords:** Single-cell DNA sequencing, simulator, DNA sequencing, Hierarchical Dirichlet

## Abstract

**Background:**

Recently, it has become possible to collect next-generation DNA sequencing data sets that are composed of multiple samples from multiple biological units where each of these samples may be from a single cell or bulk tissue. Yet, there does not yet exist a tool for simulating DNA sequencing data from such a nested sampling arrangement with single-cell and bulk samples so that developers of analysis methods can assess accuracy and precision.

**Results:**

We have developed a tool that simulates DNA sequencing data from hierarchically grouped (correlated) samples where each sample is designated bulk or single-cell. Our tool uses a simple configuration file to define the experimental arrangement and can be integrated into software pipelines for testing of variant callers or other genomic tools.

**Conclusions:**

The DNA sequencing data generated by our simulator is representative of real data and integrates seamlessly with standard downstream analysis tools.

## Background

Simulation software is important for developing and improving statistical methodology for next-generation sequencing data [1]. There are currently 149 such genetic data simulators indexed by the National Cancer Institute [2], and four of these simulators produce DNA sequencing reads with single-nucleotide variants: GemSIM [3], NEAT [4], SInC [5], and CuReSim [6]. Huang et al. [7] proposed one of the first next-generation sequencing (NGS) simulators, but this simulator only generates bulk sequencing data. Gourlé et. al. [8] developed a simulator specifically for metagenomic sequencing experiments. In the past year, two novel simulators for NGS DNA sequencing data have been proposed. One tool incorporates human population genetic information to simulate structural variation and different types of nucleotide variants [9]. Another tool aims to simulate data from single-cells incorporating allelic dropout, but not false positives or different types of nucleotide variants [10].

The clonal structure of a given sample is difficult to assess from single-cell data alone. Single-cell data suffers from issues like uneven sequencing and partial genome recovery and it is often too expensive to sequence enough single cells to gain a representative sample of the population. On the other hand, bulk samples effectively average over the fine-grain structure present at single-cell level. Developing methods to resolve the clonal populations and their genotypes and determine the relationships between those populations may be advanced by drawing inferences jointly from single-cell and bulk data.

A significant portion of the total sequencing data in existence is generated from experimental studies with model organisms or from repeated measurements of patient samples. These studies have two aspects that are not addressed by existing NGS simulators. First, these datasets contain sequencing data from both bulk tissue or culture as well as single cells. Second, a hierarchical study design induces correlation between samples [11, 12]. For example, an individual cancer patient is sampled from a population, then a tumor is sampled from the individual, and finally, a biopsy is sampled from the tumor. None of the aforementioned simulation tools address these aspects of real datasets.

To address this need, we have developed a software package, single-cell NGS simulator (SCSIM), to allow researchers to simulate bulk and single-cell NGS data from a hierarchical grouped sampling design.

## Implementation

**Fig. 1:**
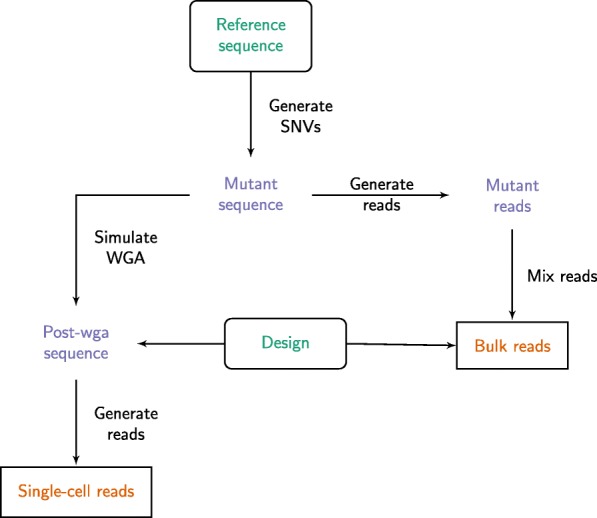
SCSIM simulation workflow. Inputs (shown in rounded boxes with green text) are the reference sequence and experiment design. Outputs (shown in cornered boxes with orange text) are the bulk and single cell FASTQ read files. Intermediate objects are shown in purple with no boxes

Figure 1 shows a high level workflow diagram of the simulator. The command-line software takes a single haploid reference sequence in FASTA format and a YAML configuration file, and produces FASTQ reads that can be used for downstream alignment and variant calling tasks. The source code can be downloaded from the github repository https://github.com/flahertylab/scsim and the implementation can be run from a docker container defined in the repository.

The nested sampling structure is implemented using a truncated hierarchical Dirichlet mixture model. This model is related to the hierarchical Dirichlet process mixture model in that if the number of components *K*→*∞* and the genotypes are drawn i.i.d. from a base measure [13] then the model converges to a hierarchical Dirichlet process mixture model. In sequencing data sets, it is more common to have a fixed number of genotypes, so the Dirichlet mixture model is implemented for this simulator. Errors induced by whole-genome amplification (WGA) of single-cells are simulated using the method described by Zafar et. al. [14]. Then, given the set of diploid reference sequences, NGS reads are simulated using dwgsim [15]. Finally, bulk NGS data is simulated by sampling without replacement from the set of reads from pure samples in proportions defined by the hierarchical Dirichlet model.

### Mutated synthetic prototype genome simulation

Mutated diploid sequences are generated from a single reference (FASTA). Given *K*—the number of mutated synthetic prototype genomes and *n*—the number of possible SNV locations, one-third of SNVs are shared across all mutated sequences, one-third of SNVs are shared across one-half of the mutated sequences, and the remaining one-third of SNVs are shared across a proportion of sequences chosen from a uniform distribution [14]. The set of mutated synthetic prototype genomes is represented by ***H*** where ***h***_*k*_ is a vector of length *n* containing the mutation location and type information for the *k*^*th*^ mutant genome. The locations of the SNVs are equally spaced across the region of interest in the reference FASTA. Given the status of each SNV location in each mutated sequence, the type of diploid mutation (heterozygous or homozygous) and base substitutions are generated according to a transition probability matrix derived from Pattnaik et. al. (SiNC) [5]. The transition probabilities, and SNV locations can be set by the user, and have default values derived from literature.

### Hierarchical sampling model

We define a *biological unit* as the top-level sampling unit. For example, in cancer sequencing a biological unit may be an individual patient, or, in experimental model organism sequencing, a biological unit may be a biological replicate. A *sample* is the bottom-level sampling unit. In cancer sequencing, a sample may be a single-cell or bulk biopsy, or, in experimental model organism sequencing, a sample may be a technical replicate.

A hierarchical Dirichlet model is used to simulate correlation between samples. First, the population distribution over mutated synthetic prototype genomes, ***G***^***″***^, is sampled from a Dirichlet distribution with parameter ***α***. Then, the distribution over mutated sequences for biological unit *i*, ***G***^***′***^_*i*_, is sampled from a Dirichlet distribution with parameter *β*_*i*_***G***^***″***^ where *β*_*i*_ controls the concentration of the distribution of the biological unit around the population distribution. Finally, the distribution over mutated sequences for sample *j* in biological unit *i*, ***G***^***′***^_*ij*_, is distributed as a Dirichlet with parameter *γ*_*ij*_***G***^***′***^_*i*_ where *γ*_*ij*_ controls the concentration of the sample around the biological unit distribution. The distributions, ***G***^***″***^, ***G***^***′***^_*i*_, and ***G***^***′***^_*ij*_ are all *K*−dimensional vectors because there are *K* mutant synthetic genomes in ***H***.

**Fig. 2:**
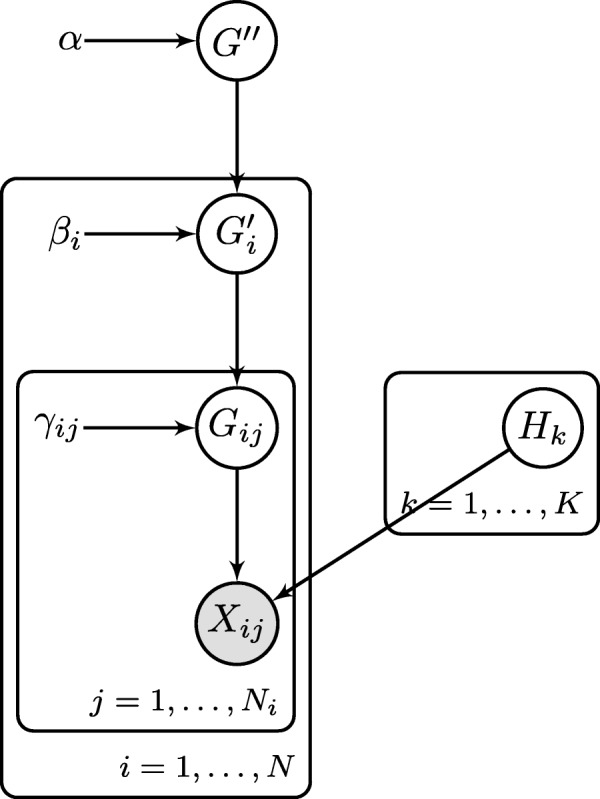
Graphical model representation of the SCSIM hierarchical model. To generate data from the model, first, sample a distribution over *K* mutant genomes, ***G***^″^. Sample a distribution for each biological unit (individual), ***G****i*′ for *i*=1,…,*N*. Sample a distribution for each sample (biopsy), ***G****ij*′ for *j*=1,…,*N*_*i*_. Sample variant locations and types that define the mutant synthetic genomes ***H***=[***h***_1_,…,***h***_*K*_]. Finally, sample the bulk and single-cell reads for each sample ***X***_*ij*_

The hierarchical Dirichlet generative model is summarized as

1a $$\begin{array}{*{20}l} \boldsymbol{G^{\prime\prime}} &\sim \text{Dirichlet}(\boldsymbol{\alpha}),& \end{array} $$

1b $$\begin{array}{*{20}l} \boldsymbol{G^{\prime}}_{i} &\sim \text{Dirichlet}(\beta_{i} \boldsymbol{G^{\prime\prime}}), &{for}\ i=1, \ldots, N \end{array} $$

1c $$\begin{array}{*{20}l} \boldsymbol{G}_{ij} &\sim \text{Dirichlet}(\gamma_{ij} \boldsymbol{G'}_{i}), &{for}\ i=1, \ldots, N,\quad j=1, \ldots, N_{i} \end{array} $$

1d $$\begin{array}{*{20}l} \boldsymbol{H}_{k} &\sim \textrm{SiNC}(\theta), &\text{for}\ k=1, \ldots, K \end{array} $$

where SiNC is the model for generating variant locations and types which depends on parameter *θ* [5]. Figure 2 shows a graphical model representation of Model 1 where ***X***_*ij*_ are the reads generated according to the bulk and single-cell sampling models.

To ground the sampling model in a real data example, we consider a cancer sequencing dataset. First, a distribution over cancer genotypes is sampled from ***G***^***′′***^. Then, for each individual patient (biological unit), *i*, a distribution over cancer genotypes, ***G***^***′***^_*i*_, is sampled. This distribution represents the fraction of the total tumor burden in the individual from each clonal genotype. Finally, for each biopsy (sample), *j*, a distribution over cancer genotypes, ***G***_*ij*_, is sampled. This distribution represents the fraction of the bulk or single-cell biopsy from each clonal genotype. Clearly, the distribution over genotypes for single-cell biopsy should be concentrated at a single genotype; whereas the distribution for a bulk biopsy may be concentrated at a single genotype or more diffuse if the biopsy is heterogeneous. The hierarchical Dirichlet model allows our simulator to generate data from exactly this experimental sampling structure.

### Sampling models

The observed reads for sample (*i*,*j*), ***X***_*ij*_, are generated differently for bulk and single-cell samples. The total number of samples for biological unit *i* is the summation of the single-cell samples and the bulk samples, $N_{i} = N_{i}^{\texttt {sc}} + N_{i}^{\texttt {bu}}$. We describe the single-cell and bulk read sampling models next.

**Single-cell data sampling model** For each of the $N_{i}^{\texttt {sc}}$ single-cell samples, a whole-genome amplification (WGA) model is applied to the corresponding mutated sequence. Allelic dropout (ADO) and false positive (FP) mutations from the WGA process were generated as done previously [14]. The ADO rate was set to 20% and the FP rate was set to 3.2×10^−5^ [16]. The ADO and FP rates are calculated in reference to the entire length of the reference sequence. Finally, sequencing reads and corresponding FASTQ files were generated with dwgsim for each of the *N*_*sc*_ single-cell samples.

**Bulk data sampling model** First dwgsim was used to generate FASTQ reads for each of the *K* mutated synthetic prototype genomes. Then for each of the $N_{i}^{\texttt {bu}}$ bulk samples, the FASTQ reads from each of the mutated synthetic prototype genomes were mixed according to the sample distribution *G*_*ij*_.

## Results

Here we test whether the output generated by SCSIM are consistent with the output generated by real experiments. We say that the output is consistent when the FASTQ read files generated by SCSIM produce variant calls that are comparable to variant calls produced from FASTQ files from real experiments.

**Simulation protocol** In order to assess the consistency of reads simulated by SCSIM, we used the results from two variant callers: BCFtools [17] and Monovar [14]. We measure the accuracy of the variant callers in terms of (1) true positive rate or recall and (2) positive predictive value or precision.

**Fig. 3:**
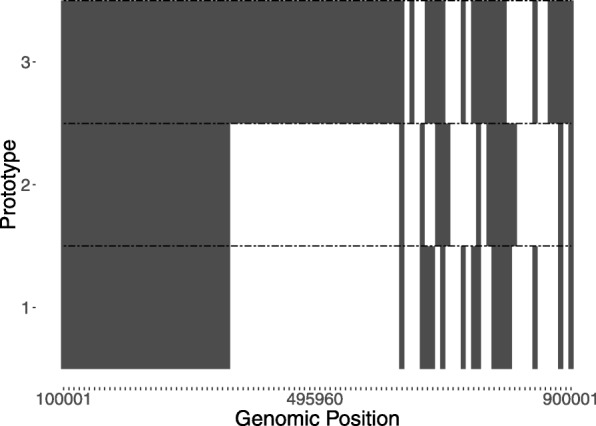
Simulated true SNV locations across mutated synthetic prototype genomes

We extracted a 1 million base pair region from hg38 starting at chr20:100000. We generated three diploid synthetic prototype genomes each with 100 potential SNVs spaced every 8080 base pairs. The zygosity of the SNVs was sampled according to the method described in the “Implementation” section. The prior parameter for the Dirichlet distribution across mutated synthetic prototype genomes, *α* was set to (0.1,0.3,0.6). Figure 3 shows the distribution of true SNVs across genomic position for each mutated synthetic prototype genome. The concentration parameter for the Dirichlet distribution at the biological unit level *β*_*i*_, was set to 0.1 for all *i*. The concentration parameter for the Dirichlet distribution for samples within biological units, *γ*_*ij*_ was set to 0.1 for all (*i*,*j*). Two samples were generated for each unit with unit 1 and 4 having one bulk and one single-cell sample, unit 2 having two single-cell samples, and unit 3 having 2 bulk samples. The mean coverage level was set to 24 ×. For bulk samples, we drew 1,000,000 reads according to the distribution over prototypes that was realized by the hierarchical Dirichlet model. A total of 90 SNVs introduced across 4 single-cell and 4 bulk samples from the described simulation protocol are the true SNVs and served as the gold standard set.

**Fig. 4:**
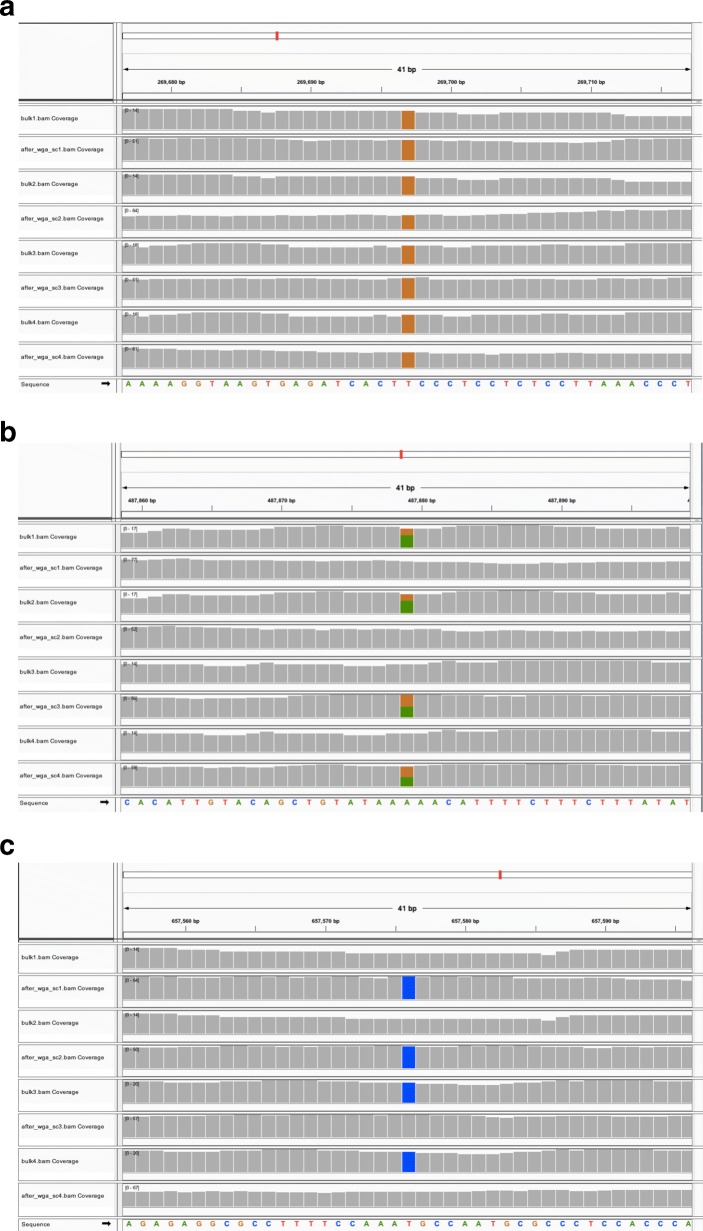
IGV visualization of reads generated by SCSIM for 4 single-cell and 4 bulk samples at **a** a genomic location where each sample has a true SNV, **b** at a genomic location where half of the samples have a true SNV, and **c** at a genomic location where a random fraction of the samples have a true SNV

**Visualization of simulated reads** The simulated FASTQ reads of single-cell and bulk samples across 4 biological units were mapped to the human genome assembly (hg38) using the Burrows-Wheeler alignment tool [18]. Figure 4 shows IGV visualizations of three genomic locations with varying proportions of samples with true SNVs.

We compared the quality metrics of SNVs detected from simulated reads to the four different human acute lymphoblastic leukemia patients that were previously published [19]. That study collected both single-cells and bulk samples of six patients to better understand genomic heterogeneity. A 1-Mbp region of chromosome 20 of the human genome (hg38) was chosen as the reference genome. Both simulated reads and the patient data were aligned and variants were called on 1-Mbp region. The Ti/Tv (transition to transversion) ratio of both simulated and patient data fall within the range of 1.2–1.6. We also compared substitutions percentages across the simulated reads and the patient data. While most substitutions of the patient and simulated data fall within the comparable range, less than expected T >A substitutions in our patient data and C >A in the simulated data. This can be attributed to the nature of single-cell data which suffers from issues like uneven sequencing, coverage, and partial genome recovery.

Mapped reads were used to call variants by Monovar [14] and BCFtools [17], two popular SNV callers used for the single-cell data. Monovar and BCFtools were run with default parameter values on the BAM files of all single-cell and bulk data.

**Fig. 5:**
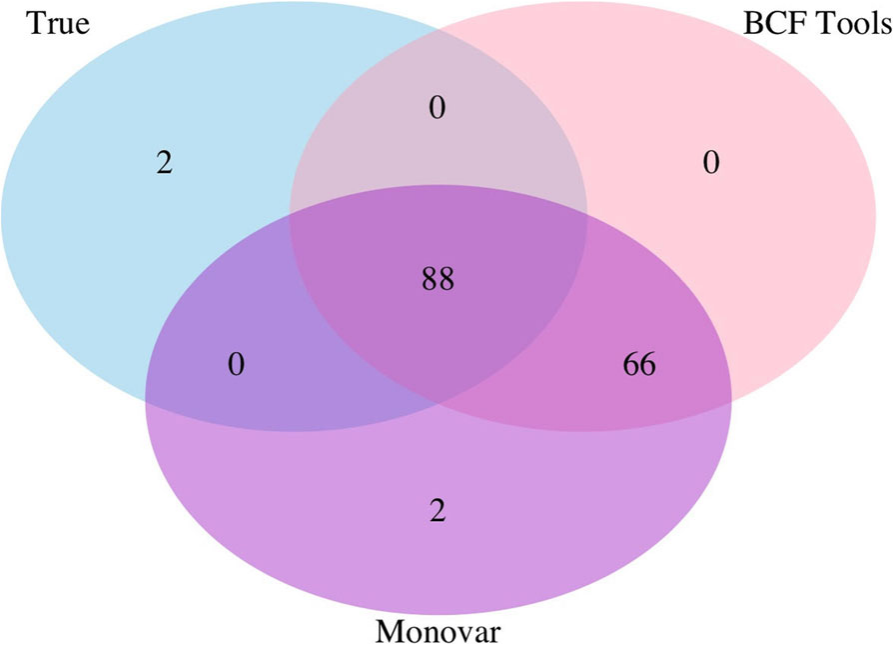
Venn diagram showing concordance of called and true variants

BCFtools and Monovar called 154 and 156 SNVs respectively across 4 single-cell and 4 bulk simulated samples. Our analysis showed that out of 90 true SNVs, 88 were called by both BCFtools and Monovar resulting in a recall of 97.77% for both methods. BCFtools had a precision of 57.1% and Monovar had a precision of 56.4%. Previous research has shown that variant calls from BCFtools and Monovar tend to have high sensitivity and low specificity (see [14] Supplementary Figure 3 and [20]). Low specificity may be acceptable if the variant calls are heavily filtered to reduce the final false positive rate. Figure 5 shows a Venn diagram of the concordance between true SNVs and called SNVs from BCFtools and Monovar.

It is common to post-filter the variant calls to increase specificity at the expense of decreased sensitivity. We applied the following standard post-filtration steps to the variant calls from Monovar and BCFtools: (1) minimum mapping quality of reads (>1), (2) minimum base quality (>30), (3) minimum number of variant-supporting reads (>5), (4) remove strand biasedness. As expected, after filtration, recall decreased, and precision increased. The F1-score, a weighted measure of precision and recall, for both Monovar and BCFtools is 0.779. In comparison, the F1-score before filtration is 0.715 for Monovar and 0.725 for BCFtools. These F1-scores are in-line with previously reported analysis on real and simulated data which is consistent with the total number of reads across all samples indicating that our simulation tool provides FASTQ reads consistent with real data [14, 21].

## Conclusions

Given the increase in the amount of single-cell next-generation DNA sequencing data there is a need for reproducible bioinformatics methods for performing statistical inference on that data. To our knowledge there are no methods for jointly simulating bulk and single-cell sequencing data, yet these simulation tools are needed to test and validate inference methods. SCSIM jointly simulates bulk and single-cell next-generation sequencing data and generates correlated samples using a hierarchical truncated Dirichlet distribution for sampling the distribution over mutant sequences for bulk samples. Our implementation, using a docker container, allows it to be inserted in a bioinformatics pipeline without modifying existing dependencies.

## Availability and requirements

**Project name**: single-cell DNA sequencing data simulator

**Project home page**: https://github.com/flahertylab/scsim

**Operating systems(s)**: Any

**Programming language**: Python

**Other requirements**: docker

**License**: MIT

**Any restrictions to use by non-academics**: none

## Data Availability

The data and materials used in this study are openly available at the locations described in the relevant sections.

## Abbreviations

ADO: allelic dropout
FP: false positive
SCSIM: single-cell NGS simulator
NGS: next-generation sequencing
WGA: whole-genome amplification
